# A Circular to the Medical Profession

**Published:** 1874-01

**Authors:** 


### To the Medical Profession at Large!

In obedience to the resolution of the Convention of Physicians, lately assembled in this city, we undertake this address to your sympathy, with full assurance that its hallowed object will secure your cordial support.

But yesterday our community was as a house of mourning, and its wail of distress, arising from stricken hearts and desolate homes, smote the ear of the world with the horrors of our affliction. Those of our people who remained in the doomed city can alone appreciate the sad story of suffering seen and felt in our midst, when death thus held, for seven long weeks, its high carnival among us. Over the entire city death and silence brooded. Its deserted streets, alike both day and night, scarce echoed a sound save the mournful hearse-rattle as it hurried to the grave its load of dead, or the foot-falls of those ministers of mercy who lighted up the hours of darkness with their visits of charity. Silently and continually the pious labor of love was performed, and each rivaled the other in the patient discharge of a common humanity. When the shaft of death prostrated one, another with true Corsican spirit took his place, and the work of benevolence went fearlessly on, until under the favor of Heaven the disease was baffled and the reign of terror at an end. Each creed, sect, order, and brotherhood had its heroes and its martyrs, and it is in commemoration of the deeds of both the living and the dead that our hearts should never suffer forgetfulness.

The faithful physician who survived the storm bears in his conscience its plaudits of duty done; but our seven brothers, Williams, Freeman, Crone, Hatch, Kennon, Blount, and Minor, fallen at their post, martyrs to the cause of humanity, aye bright exemplars of professional honor and duty, sleep in their quiet graves, with more lasting glory than embalmed warrious in piles of storied marble. Our fallen Brothers, if they could be consulted, would doubtless wish no fitter burial than quiet interment in leafy Elmwood, but professional pride demands the honor of their perpetual commemoration, and we ask in this behalf that suitable stone be raised and carved for them. Their fame, the story of their heroism, belongs to the medical world, and our brethren throughout the broad land are respectfully requested to contribute something to this laudable end. Your contributions, however small, will aggregate a success of the enterprise.

Remittances may be made to either member of the Committee.

Richard H. Taylor, M. D., 44 North Court St.
F. L. Sim, M. D., 115½ Beale St.
R. W. Mitchell, M. D., 275 Main St.

Memphis, Tenn., Nov. 10, 1873.

